# Indirect to Direct Charge Transfer Transition in Plasmon‐Enabled CO_2_ Photoreduction

**DOI:** 10.1002/advs.202102978

**Published:** 2021-11-12

**Authors:** Yimin Zhang, Lei Yan, Mengxue Guan, Daqiang Chen, Zhe Xu, Haizhong Guo, Shiqi Hu, Shengjie Zhang, Xinbao Liu, Zhengxiao Guo, Shunfang Li, Sheng Meng

**Affiliations:** ^1^ Key Laboratory of Material Physics Ministry of Education School of Physics and Microelectronics Zhengzhou University Zhengzhou 450001 P. R. China; ^2^ Beijing National Laboratory for Condensed Matter Physics and Institute of Physics Chinese Academy of Sciences Beijing 100190 P. R. China; ^3^ School of Physical Sciences University of Chinese Academy of Sciences Beijing 100190 P. R. China; ^4^ School of Physics and Information Technology Shaanxi Normal University Xi'an 710119 P. R. China; ^5^ Departments of Chemistry and Mechanical Engineering The University of Hong Kong Hong Kong 999077 P. R. China; ^6^ HKU Zhejiang Institute of Research and Innovation The University of Hong Kong Hangzhou 311305 P. R. China

**Keywords:** CO2 photoreduction, direct charge transfer, indirect hot electron transfer, plasmon‐enabled photocatalysis, time‐dependent density functional theory

## Abstract

Understanding hot carrier dynamics between plasmonic nanomaterials and its adsorbate is of great importance for plasmon‐enhanced photoelectronic processes such as photocatalysis, optical sensing and spectroscopic analysis. However, it is often challenging to identify specific dominant mechanisms for a given process because of the complex pathways and ultrafast interactive dynamics of the photoelectrons. Here, using CO_2_ reduction as an example, the underlying mechanisms of plasmon‐driven catalysis at the single‐molecule level using time‐dependent density functional theory calculations is clearly probed. The CO_2_ molecule adsorbed on two typical nanoclusters, Ag_20_ and Ag_147_, is photoreduced by optically excited plasmon, accompanied by the excitation of asymmetric stretching and bending modes of CO_2_. A nonlinear relationship has been identified between laser intensity and reaction rate, demonstrating a synergic interplay and transition from indirect hot‐electron transfer to direct charge transfer, enacted by strong localized surface plasmons. These findings offer new insights for CO_2_ photoreduction and for the design of effective pathways toward highly efficient plasmon‐mediated photocatalysis.

## Graphical abstract

Upon illumination, hot electrons generated by the plasmon decay around the silver clusters transfer into the molecular orbitals of CO_2_, resulting in activation of the asymmetrical stretching and bending mode of CO_2_ and leading to its final splitting. Cooperative interaction and transition from indirect hot‐electron transfer to direct charge transfer cause the nonlinear relationship between laser intensity and reaction rate.

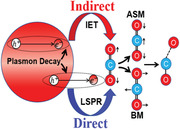

## 1 Introduction

Localized surface plasmon resonance (LSPR) is the collective oscillation of electrons in metal nanostructures induced by photoexcitation, which usually exhibits distinct properties such as extended lifetime, tunable resonance energy, ultrafast dynamics and enhanced optical absorption.^[^ ^1^, ^2^, ^3^ ^]^ These properties enable plasmonic nanostructures to serve as excellent platforms for the exploration of light‐matter interactions in a wide range of applications such as photodetection,^[^ ^4^, ^5^ ^]^ photovoltaics,^[^ ^6^ ^]^ surface‐enhanced Raman spectroscopy,^[^ ^7^, ^8^, ^9^ ^]^ photoelectrochemistry^[^ ^10^ ^]^ and photocatalysis.^[^ ^11^, ^12^, ^13^ ^]^ In particular, plasmon‐induced photocatalytic reactions of molecules adsorbed on metal nanostructures have sparked considerable attention in chemical and solar energy conversions in the last decade.^[^ ^14^, ^15^, ^16^, ^17^, ^18^, ^19^, ^20^, ^21^ ^]^ However, such processes suffer from slow kinetics and low conversion efficiency, due to the complex interactive dynamics of the hot carriers between plasmonic nanomaterials and the adsorbates. It is therefore highly important to clarify the underlying catalytic mechanisms at the electronic structural level, so as to identify the energy dissipation pathways and thus improve effectively the photocatalytic performance. Moreover, due to its relatively high inertness and large presence as a waste greenhouse gas, chemical conversion of carbon dioxide (CO_2_) is very challenging but highly significant both scientifically and socioeconomically. Plasmon‐induced photoreduction of CO_2_ offers a potentially effective route to enhance CO_2_ conversion and utilization, and hence the process is selected here for focused study.

**Figure 1:**
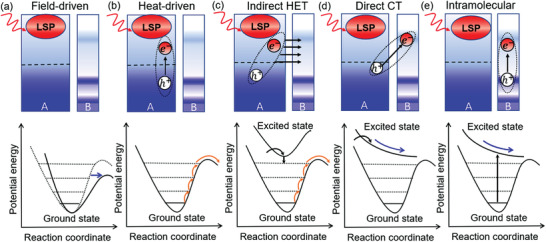
Schematic illustration of different mechanistic models of plasmon‐induced photocatalysis. The bottom panels denote the corresponding potential energy surfaces (PES) of adsorbates. a) Plasmon‐field‐driven mechanism. b) Plasmon heat‐driven (thermal‐field‐driven) mechanism. c) Indirect hot‐electron transfer (HET) mechanism. d) Direct charge transfer (CT) mechanism. e) Intramolecular charge transfer mechanism. A: Metal, B: Adsorbate. The dashed line in the top panels denotes the Fermi level, and the dotted lines in the bottom panels denote vibrational energy levels. Orange and black arrows represent transitions among vibrational levels and the electron transfer, respectively, while the blue arrows represent the flattening and reduction of PES, respectively.

For plasmon‐induced catalysis, several reaction mechanisms have been proposed in the past, based on experimental or theoretical reasoning, which are schematically summarized in **Figure** **1**: a) Plasmon‐field‐driven catalysis (instantaneously, timescale ≈10 fs),^[^ ^22^, ^23^, ^24^, ^25^ ^]^ b) Plasmon‐assisted heat‐driven catalysis (with a typical timescale ≈100 ps to 10 ns),^[^ ^26^, ^27^, ^28^ ^]^ c) indirect hot‐carrier transfer catalysis (≈100 fs),^[^ ^11^, ^29^, ^30^, ^31^, ^32^, ^33^, ^34^, ^35^ ^]^ d) direct charge transfer catalysis (≈5 to 100 fs),^[^ ^36^, ^37^, ^38^, ^39^, ^40^ ^]^ and e) intramolecular charge transfer catalysis (≈5–20 fs).^[^ ^41^, ^42^ ^]^ These mechanisms are well distinguished by their active timescales.^[^ ^43^, ^44^ ^]^ For plasmon field driven catalytic process, the plasmon field generated after laser illumination can drag and then flatten the potential energy surface (PES) of ground state as shown in Figure 1a. Given that the plasmon filed could flatten the PES rather than introducing electronic excitations, the system will not be excited to the excited‐state PES but simply remains in the ground state. Therefore the ground‐state PES simply changes its shape (being flattened) under the plasmon field and the energy minimum would not change significantly. Once the plasmon field could strongly flatten the PES, the barrier of the reaction process will become extremely small so that the reaction process could take place easily while the system remains in the ground state. The plasmon‐field‐driven reaction considers the overall “near‐field” effect of the LSPR, which describes well of the field enhancement phenomena by plasmons, but cannot adequately consider quantum dynamic effects that dominate small systems of ≈1 nm. Furthermore, the aggregation of metal particles results in the formation of electromagnetic hot spots, which can significantly enlarge the field enhancement. The heat‐driven model only considers the local thermal field around the nanoclusters generated by plasmon decay via electron‐electron, electron‐phonon and phonon–phonon interactions, which again mainly involves the thermal effects in relatively long spatial and temporal scales. Moreover, only when the phonon modes of the nanostructures are coupled with the vibrational modes of the reactants at relatively high intensities, the transformation of the adsorbate from the reactant to the product state can be effective, as noted from the ground‐state potential energy surface (PES) analysis.^[^ ^27^ ^]^ However, reliance of such processes generally requires relatively high temperatures, which is inefficient for catalysis.

For the indirect hot‐electron transfer (HET) mechanism, the hot electrons of varied energy distribution above the Fermi level, originated from nonradiative plasmon decay, can be injected into the adsorbate via inelastic electron tunneling (IET), forming a transient negative ion (TNI) of the absorbed molecule and subsequently leading to molecular reduction via vibrational transitions.^[^ ^32^, ^35^ ^]^ However, most of the hot electrons are close to the Fermi level, i.e., only the orbitals near the Fermi level are preferentially involved, which result in limited catalytic efficiency. For the direct charge transfer (CT) mechanism, electrons at a specific energy level can be directly transferred to an unoccupied orbital of the adsorbate‐metal complex, forming holes in the metal and a TNI state of the adsorbate simultaneously. This process reduces the energy loss of the excited electrons due to the lack of electron‐electron scatterings and thus can significantly enhance chemical selectivity.^[^ ^36^ ^]^ Because of the relatively small transition dipole moments in the adsorbate‐metal complex, the direct charge transfer is usually negligible unless in the presence of a strong field, and so does the intramolecular charge transfer mechanism, which requires the hybridization of electronic states between the absorbed molecule and the metal.^[^ ^41^, ^42^ ^]^ Evidently, it is critically important to clarify which of the above mechanisms is the most effective and whether different mechanisms may contribute synergistically to the plasmon‐mediated processes, particularly for the practically important and chemically inactive CO_2_ molecule.

**Figure 2:**
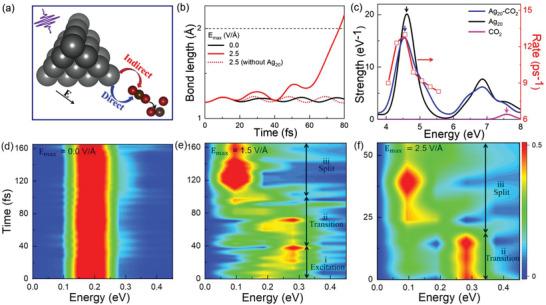
Dynamic responses of plasmon excitation. a) Schematic of plasmon‐induced CO_2_ reduction on Ag_20_ (tetrahedron) upon a laser field polarized in the *x*‐direction (parallel to the CO_2_) along with the molecule. b) Time‐evolved C═O bond length under different conditions. The black dashed line denotes the bond breaking at 2.0 Å. c) Absorption spectra of Ag_20_‐CO_2,_ Ag_20_, and CO_2,_ respectively, and the red line denotes the calculated reaction rate with different levels of photon energy. Vibrational modes of the CO_2_ species obtained via Fourier transform of the C═O bond length of d) a freestanding CO_2_ molecule without light (*E*_max_ = 0.0 V Å^−1^), and CO_2_ adsorbed on Ag_20_ with e) *E*_max_ = 1.5 V Å^−1^ and f) *E*_max_ = 2.5 V Å^−1^, respectively. Here, the color bar denotes the relative strength of vibration energy.

Here from the perspective of the atomic and electronic spatial and temporal (femtosecond) scales, we investigate the underlying carrier dynamics of plasmon‐induced CO_2_ reduction on plasmonic nanostructures with different sizes and geometries using time‐dependent density functional theory (TDDFT), represented by typical tetrahedral Ag_20_ and icosahedral Ag_147_ magic clusters. As shown in **Figure** **2**a, a synergic interplay is revealed between the indirect HET and direct CT mechanisms, which breaks the linear dependence of the reaction rate on the laser intensity for systems where only a single reaction mechanism is involved. We further identify that the photoresponse can be divided into distinct regimes controlled by different mechanisms. More specifically, hot electrons originated from plasmon decay are prone to migrating toward the lowest unoccupied molecular orbital (LUMO) of CO_2_ via IET, whereas direct charge transfers into high‐lying LUMO+11 and LUMO+12 states (for Ag_20_‐CO_2_) are also observed; these synergistically create the TNI state of CO_2_ (CO_2_^−^) and facilitate photoreduction. During the photoreduction process, asymmetric stretch mode (ASM) and the bending mode (BM) of CO_2_ are clearly discerned. These findings provide strong evidence that different pathways could synergistically enhance photocatalytic reactions, which paves the way toward the design of more effective and selective plasmonic photocatalysts.

## 2 Results and Discussion

### 2.1 The Response of Plasmon Excitation

To clarify the mechanisms of the interaction, we first investigate the dynamic response of CO_2_ on a representative nanocluster Ag_20_ upon a laser pulse of a maximum field strength *E*_max_ = 2.5 V Å^−1^ with the corresponding photon energy *ℏω* = 4.56 eV, where *ω/2π* is the light frequency (see the Experimental Section and Figure S1a, Supporting Information). The time‐dependent changes in the bond length of C═O are shown in Figure 2b. It is noted that the frequency of the applied laser pulse matches well with the plasmonic absorption spectra calculated for the tetrahedral Ag_20._^[^ ^45^ ^]^ Specifically, we observe that the O‐CO distance is gradually extended, though with significant oscillation. According to previous reports, when the O‐CO distance is larger than 2.0 Å, the CO_2_ molecule can be regarded as being split without significant interactions between the split O and CO species.^[^ ^46^, ^47^ ^]^ For simplicity, we define the rate of the CO_2_ splitting as the inverse of time when the length of the first C═O bond reaches 2.0 Å. Besides, the products stay in oscillation in the gas phase instead of being adsorbed on the Ag_20_ cluster (Figure S1b, Supporting Information), excluding the issue of oxidation and CO poisoning. The CO_2_ molecule splits at 77 fs with the length of C═O bond increases from 1.17 to 2.0 Å. In addition, as shown in Figure 2b, the oscillation period increases from 17 to 20 fs, which implies that the molecule is activated from the ground state to the excited states upon laser illumination.

To elucidate further the effect of the Ag cluster, we also explored the dynamic response of an isolated CO_2_ molecule to the laser pulse without the Ag nanostructure. The O‐CO distance of the gas‐phase CO_2_ molecule oscillates around 1.17 Å, rather than being split in the same laser field. These results indicate clearly that the CO_2_ reduction is mainly driven by plasmonic excitation in the Ag nanostructure.

To illustrate the dependence of the CO_2_ reduction on the photon energy, we show the absorption spectra of a freestanding CO_2_ molecule, Ag_20_, and the Ag_20_‐CO_2_ complex in Figure 2c, respectively. The absorption peak of the freestanding CO_2_ is at 7.63 eV, corresponding to the photon‐excited electron transition from the highest occupied molecular orbital (HOMO) to the lowest unoccupied molecular orbital (LUMO). The Ag_20_‐CO_2_ complex shows a visible red shift relative to Ag_20_, indicating a non‐negligible coupling between CO_2_ and Ag_20_. Here, according to relative high absorption strength, the first peak 4.56 eV is chosen for analysis. In addition, with the corresponding increase of the photon energy from 4.06 to 5.31 eV for a given pulse, as parameterized in Figure 2c, the rate of the C═O bond breaking reaches the maximum when the photon energy equals the resonance energy of the absorption peak. These findings further confirm that the CO_2_ reduction is largely induced by plasmon excitation over the Ag cluster.

### 2.2 Excitation of Vibrational Modes

Furthermore, the vibrational modes of CO_2_ were analyzed, as shown in Figure 2d–f. To clarify the time‐dependent vibrational modes, we perform a fast Fourier transform on the bond length (Figure S3a, Supporting Information)

(1) $$I\left(\omega ,t\right)={\left|\int_{t}^{t+T}l\left(t^{\prime }\right)e^{-i\omega t^{\prime }}dt^{\prime }\right|}^{2}$$

where *l*(*t’*) is the C═O bond length at time *t’, T* is the time window, *I*(*ω*, *t*) is the vibration intensity of frequency *ω* during time *t ∼ t + T*. Taking the case of *E*_max_ = 1.5 V Å^−1^ as an example, the CO_2_ split process can be qualitatively divided into three steps: i) vibrational excitation (0∼40 fs): it is observed that upon the laser illumination the vibrational peak located initially at 0.18 eV, i.e., the symmetric stretching mode (SSM), is gradually blue‐shifted to 0.29 eV, corresponding to the asymmetric stretching mode (ASM); ii) transition (40∼100 fs): the dominant vibrational peak is now located around the ASM, implying a short‐lived transition state; and iii) split (100∼160 fs): the ASM located at 0.29 eV is in turn significantly red‐shifted to a transient state located at 0.09 eV, which corresponds to the largely extended C═O bond length or the splitting of the CO_2_ into an O and a CO species. Moreover, it is observed that the time scales of all the three steps are dramatically reduced when *E*_max_ is increased from 1.0 to 2.5 V Å^−1^, see Figure S3b,c (Supporting Information). In experiments,^[^ ^48^ ^]^ the vibrational frequency for the reactant and product species during the reaction process can be detected using in situ single‐NP SERS spectroscopy. The experimental frequency (1421 cm^–1^ corresponding to the Fermi resonant *ν*_c+_ mode) is in close agreement with our simulations (0.18 eV or 1451 cm^–1^) for the C═O vibration in CO_2_ in the initial stage. We also explored the time evolution of the bond angle ∠O–C–O, as shown in Figure S1c (Supporting Information). With the increase of the *E*_max_, the bond angle decreases from 180° to ∼90°, which is usually accompanied by a strong activation of bending mode (BM) or the split of the CO_2_ molecule via charge transfer processes.^[^ ^49^, ^50^, ^51^ ^]^ Particularly, the excitation of ASM and BM (Figure S1c, Supporting Information) is unexceptionally observed_,_ which implies that they are indispensable for the photoreduction of CO_2_.

### 2.3 Field Enhancement and Charge Transfer Δ*Q*

**Figure 3:**
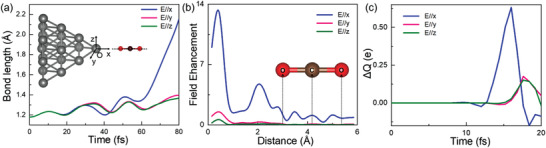
The polarization‐dependent responses. a) Time evolution of C═O bond length in three polarized directions, *x*, *y*, *z*, respectively. The inset shows the atomic geometry of the system. “O” denotes the origin of coordinates. b) Local field enhancement (FE) factor for the field along the *x*‐axis, and c) the accumulated charge Δ*Q* around CO_2_, with laser electric field polarized along *x*, *y*, *z* directions, respectively. The inset in (b) denotes the position of the O–C–O atoms along the *x* axis.

To uncover the dominant mechanism of CO_2_ photoreduction, we compare the time evolution characteristics of the C═O bond length under the same pulse, polarized in the three orthogonal directions, i.e., *x*, *y*, and *z*, respectively (**Figure** **3**a). In doing so, we first calculated the corresponding localized field enhancement (FE) as defined by

(2) $$\mathrm{FE}\left(x\right)=\frac{\left[V_{\mathrm{eff}}\left(x+\delta x\right)-V_{\mathrm{eff}}\left(x\right)\right]}{e\delta xE_{\mathrm{ext}}}\;$$

where *x*, *δx*, v_eff_, and *E*_ext_ are the position, mesh size along the polarized direction (*x*), effective potential and external field strength, respectively.^[^ ^52^ ^]^ As shown in Figure 3a, the CO_2_ reduction can only be observed under *E*_ext_(*x*) which are polarized along the axial direction of CO_2_. Meanwhile, an average FE of 1.7 is probed around the CO_2_ species, in close agreement with previous investigations.^[^ ^22^, ^53^ ^]^ Interestingly, since negligible FE is observed on the CO_2_ molecule when the *E*_ext_ is polarized along the y or the z direction, the average C═O bond length is only slightly enlarged, insufficient for bond breaking. Moreover, for the freestanding CO_2_ molecule under a strong pulse with *E*_max_ = 4.2 V Å^−1^, which corresponds to FE = 1.7, the C═O bond length still only oscillates between 1.15 and 1.23 Å (Figure S4, Supporting Information), ruling out purely field‐driven mechanisms. We emphasize that the temperature effect on the CO_2_ reduction induced by the heat‐driven mechanism can also be excluded here, due to its much longer time scale (≈100 ps) than that in the present case, namely, around 80 fs.

To elucidate the charge transfer mechanism, we present the time‐dependent charge transfer (Δ*Q*) to the CO_2_ molecule along the three aforementioned polarization directions as shown in Figure 3c. Here, the total charge density (Q) in a sphere with a radius of 3.32 Å centered at the C atom was integrated for the CO_2_ molecule, and then the charge transfer (Δ*Q*) from the Ag_20_ to the CO_2_ molecule is monitored as a function of time. As shown in Figure 3c, Δ*Q* increases to a maximum of 0.63 *e* at 16 fs under the pulse polarized along x direction, and only about 0.14 *e* is transferred from Ag_20_ to CO_2_ under the pulse polarized along y or z directions, implying that CO_2_ photoreduction is dominated by plasmon‐induced charge transfer.

**Figure 4:**
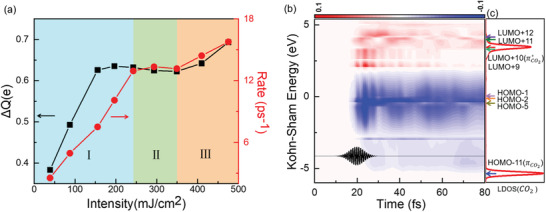
Charge transfer and time‐dependent Kohn–Sham states. a) The maximum charge transfer Δ*Q* to CO_2_ at *t* = 16 fs (black squares) and the rate of CO_2_ reduction (red circles) as a function of laser intensity with fixed photon energy of 4.56 eV. b) Time‐dependent changes in the occupation of the KS states of the Ag_20‐_CO_2_ system with a field strength of 0.1 V Å^−1^. The black curve denotes the laser pulse. The eight arrows from bottom to top denote the energy levels of the eight KS states, i.e., HOMO‐11 (*π*_CO2_), HOMO‐5, HOMO‐2, HOMO‐1, LUMO+9, LUMO+10 (π*_CO2_), LUMO+11, and LUMO+12, respectively. c) Local projected density of states (LDOS) on the HOMO (*π*) and LUMO (*π* ^*^) of CO_2_ species at *t* = 0 fs.

To correlate further the charge transfer and reduction process, we show the laser intensity‐dependent charge transfer and the corresponding reduction rate in **Figure** **4**a. Intriguingly, the dependence of the charge transfer Δ*Q* and the corresponding reduction rate exhibits significantly nonlinear features with the laser intensity, compared to a single‐mechanistic process reported experimentally^[^ ^11^, ^29^, ^32^, ^33^ ^]^ or theoretically;^[^ ^36^ ^]^ the latter only indicates a linear correlation. It is noted that the nonlinear dependence in Figure 4a can be divided into three distinct regimes. For the charge transfer in the relatively low laser intensity regime (I) of 0∼150 mJ cm^−2^, a linear correlation is observed; then, a plateau region (II) exists when the laser intensity increases from 150 to 350 mJ cm^−2^; and finally, a new linear dependence is obtained within the range (III) of 350∼470 mJ cm^−2^. Consequently, the same trend is also observed for the reaction rate (Figure 4). The above observation suggests a new mode might operate in the plasmon‐driven charge transfer and photoreduction processes.

To rationalize the nonlinear dependence above, we analyze the time evolution of the occupation of the Kohn‐Sham (KS) states, see Figure 4b. Here, the occupation of KS states is calculated by projecting the time‐dependent KS state onto the KS orbitals at time *t* = 0. It is clear that the occupancy exhibits a “sloshing” feature around the Fermi level, which results in strong oscillations in the charge density near the Ag_20_ surface.^[^ ^54^ ^]^ The population inversion of the electrons from the deep energy to the high energy states is usually the result of the plasmon's nonradiative decaying into hot electrons.^[^ ^55^, ^56^ ^]^

### 2.4 Time‐Dependent Kohn–Sham States and Charge Transfer Mechanisms

**Figure 5:**
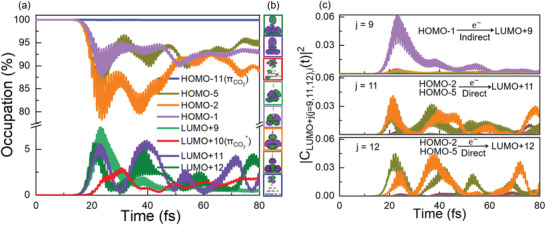
Ultrafast electron dynamics. a) Time‐evolved occupation of the eight KS states and b) the corresponding wavefunctions shown in boxes of different colors. Here, the isosurface value of the wavefunctions is 0.02 Å^–3^. c) Time‐evolved transition coefficient from all occupied states *i* to LUMO+9, LUMO+11, and LUMO+12, respectively. The index *i* (*i* = 1–18) denotes the numerical order of the occupied states, while LUMO+*j* (*j* = 0–19) corresponds to the unoccupied states. Only the important orbitals that contribute to the density change are labeled.

To determine the specific KS orbitals involved in the charge transfer, we calculated the time‐dependent occupation of the KS states for the Ag_20_‐CO_2_ complex shown in **Figure** **5**. For the sake of analysis, the weak laser pulse with a field strength of 0.1 V Å^−1^ is used. Similar results are observed under stronger laser pulses with the relative contribution of different excitation channels varies. As noted in Figure 5a, given the constant occupation of the *π*(CO_2_) orbital (HOMO−11), we can safely exclude the intramolecular charge transfer process during CO_2_ photosplitting. However, several other effects may be observed from the above results.

i) *Indirect hot‐electron transfer*: There exists significant charge transfer to the LUMO+9, which are very close to the LUMO+10 contributed mainly by the π* orbital of the CO_2_ component (see Figure 5b), enabling the indirect hot electron transfer via inelastic electron tunneling (IET) from LUMO+9 to LUMO+10. Time‐dependent transition coefficient from all the occupied states *i* to LUMO+9 demonstrates that such a charge transfer is mainly from the HOMO−1 state contributed mainly by the Ag_20_ nanocluster, see Figure 5c. These findings indicate that the charge is indirectly transferred from the Ag_20_ to the CO_2_ species via IET,^[^ ^30^, ^42^, ^57^ ^]^ as demonstrated by the significantly larger energy of the applied pulse than the (HOMO−1)−(LUMO+9) energy gap, by about 3.25 eV. Here, the photogenerated hot electrons within the metal nanoparticle transfer to the unoccupied states of adsorbates via inelastic tunneling, where an energy barrier may exist between the related orbitals. Notably, the tunneling probability can be relatively low if there exists a large energy gap between the two orbitals. So, the energy distribution of hot electrons should be close to or above the energy level of the unoccupied states of adsorbates, which facilitates inelastic electron transfer.^[30,42,54]^ In other words, the energy gap between the related orbitals would affect the electron transfer rate most significantly, while the couplings between different orbitals can be small as exhibited in the wavefunction plots in Figure 5b.

ii) *Direct charge transfer*: Meanwhile, direct charge transfer process is also identified during the photoreduction process, as noted by the strong overlap of the wavefunctions of the CO_2_ and Ag_20_ in the LUMO+11 and LUMO+12 states, as shown in Figure 5b. This direct transfer mechanism offers a new channel for plasmon decayed hot carriers, in this case electrons, to transfer directly from the Ag_20_ to CO_2_, further increasing the reduction efficiency. Indeed, the charges transferred to both LUMO+11 and LUMO+12 (located on CO_2_) is mainly from the HOMO‐5 and HOMO‐2 orbitals (located on Ag_20_), as implied by the out‐of‐phase oscillations in the occupation number between the former and the latter. Such a hypothesis is also unambiguously confirmed by the time‐dependent transition coefficient from all the occupied states *i* to LUMO+11 (LUMO+12) shown in Figure 5c. We see that except for HOMO‐5 and HOMO‐2 orbitals, all other states provide negligible contributions.

iii) *Synergetic effects and mode transition*: In previous findings of linear responses,^[^ ^11^, ^29^, ^32^, ^33^ ^]^ only a direct or an indirect charge transfer pathway is involved within the relatively low laser intensity region, and thus one can deduce that the unusual nonlinear dependence of plasmon‐induced CO_2_ reduction on laser intensity (Figure 4a) can be qualitatively explained by the synergetic role of the indirect HET and direct CT mechanisms (as shown in Figure 2a). In brief, a three‐regime model can be proposed to rationalize the electron transfer mechanisms from the present findings via TDDFT molecular dynamics (MD) simulations: For the Regime I with a relatively low laser intensity, indirect and direct charge transfer mechanisms cooperatively promote CO_2_ reduction, leading to a relatively steeper slope of linear dependency, which matches well with the experiments in the low intensity region.^[^ ^11^, ^29^, ^32^, ^33^ ^]^ For the intermediate Regime II, a plateau region for the reaction rate is reached because the two mechanisms entwine with each other and compete for photocarriers. Since the direct CT is usually 10∼50 fs faster than the indirect HET, the time mismatch results in a “back flow” of the transferred charges and the Pauli blocking of the indirect hot electrons.^[^ ^58^ ^]^ Under the extreme laser regime III, the “negative” effects of the indirect charge transfer saturate, while the increase of the charge accumulation on CO_2_ stemming from direct CT gradually takes over in determining the reduction rate. In particular, according to the difference in the slope of the reaction rate for Regimes I (0.5) and III (0.2), we can estimate the relative contribution of the two mechanisms in the three regimes: In Regime I, indirect and direct pathways contribute about 60% and 40%, respectively; for Regime II, the “negative” effect of the indirect pathway counteracts the “positive” effect of the direct pathway; for Regime III, the direct pathway dominates the reduction rate with a contribution close to 100%, while the indirect pathway is negligible.

### 2.5 General Nonlinearity and Synergetic Effects

**Figure 6:**
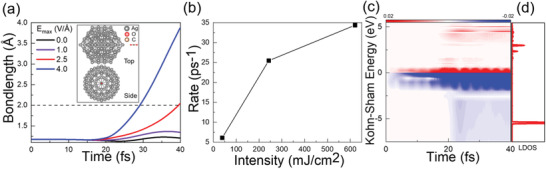
Nonlinear dynamic response of plasmon excitation of Ag_147_‐CO_2_. a) Time‐evolved bond length of C—O at *E*_max_ = 0.0, 1.0, 2.5, 4.0 V Å^−1^. The inset denotes the top and side views of the Ag_147_‐CO_2_ system. b) Rate of CO_2_ reduction as a function of laser intensity (Figure S5a,b, Supporting Information). c) Time‐dependent changes in the occupation of the KS states of the Ag_147_‐CO_2_ system with a field strength of 0.1 V Å^−1^. d) Local projected density of states (LDOS) on CO_2_ species at *t* = 0 fs.

To this end, in order to further confirm the generality of the synergetic effects and nonlinearity, we investigated the CO_2_ reduction process on other clusters with different sizes and geometries, as represented by an icosahedral Ag_147_ with a diameter of 1.64 nm (insets in **Figure** **6**a). After similar analysis of the ultrafast structure dynamics (Figure 6a,b, and Figure S5a–c, Supporting Information) and carrier dynamics (Figure 6c,d, and Figure S6a–c, Supporting Information), it is identified that the nonlinear phenomenon is also dominated by the delicate synergetic interplay between the indirect hot electron and direct charge transfer mechanisms. Hence, these results demonstrate the generality of the nonlinearity in plasmon‐mediated photocatalysis of CO_2_ reduction on Ag nanoclusters, due to the synergy of indirect hot electron and direct charge transfer.

### 2.6 Effects of the Nanoparticle Size and Adsorption Sites

The size effect can be observed directly by a twofold difference in the timescale of bond‐breaking between Ag_20_‐CO_2_ (≈80 fs as shown in Figure 2b) and Ag_147_‐CO_2_ (≈40 fs as shown in Figure 6a) with the same laser field strength *E*_max_ = 2.5 V Å^−1^, that is, the photoreduction rate is better in large clusters originated from the stronger light responses. Besides, molecular adsorption on the tip site of the nanoparticle is far more reactive than the edges and facets of the nanoparticle, toward plasmon‐induced photoreduction, since the field enhancement is the most significant on the tip sites than on nanoparticle edges and facets (details can be seen in the Supplementary Information). Similar effects are reported in Yan et al.’s work.^[^ ^22^ ^]^ In a word, relatively high intensity of laser and large nanoparticles with many tip sites are suggested for enhancing the photocatalysis process.

## 3 Conclusion

Fundamental mechanisms of plasmon‐driven CO_2_ reduction have been clarified from the perspective of ultrafast structural and electron dynamics by time‐evolved electronic occupation analysis of molecular orbitals via real‐time time‐dependent density functional theory. A CO_2_ molecule adsorbed on the tip of Ag clusters of different geometries and sizes is split by optical plasmon excitations. The reduction can be generally ascribed to the cooperative effects of indirect hot electron transfer and direct charge transfer driven by localized surface plasmon, where the relative contributions of the two mechanisms vary with the intensity of the laser. The direct charge transfer contribution increases with plasmonic (laser) intensity and eventually dominates the catalytic process. In contrast to previous findings where only direct or indirect charge transfer pathway is involved, the synergetic processes uncovered in the present work show clearly a nonlinear dependency of the reaction rate on laser intensity as observed for both Ag_20_ and Ag_147_ nanoclusters, demonstrating its generality. These findings offer new insights in the microscopic understanding of CO_2_ photoreduction, and facilitate the design of highly efficient plasmon‐mediated photocatalysts.

## 4 Experimental Section

Most of the calculations were carried out using the real‐space TDDFT code OCTOPUS,^[^ ^59^, ^60^, ^61^ ^]^ with local density approximation (LDA) for the exchange correlation functional. The simulation zone was defined by assigning a sphere around each atom with a radius of 6.0 Å and a spacing of 0.3 Å between grid points. Hartwigsen–Goedecker–Hutter pseudopotentials were used to represent the interactions between valence electrons and the atomic cores.^[^ ^62^ ^]^ Nonadiabatic molecular dynamics was treated within the Ehrenfest scheme, where the ground state was used as the initial state. A time step of 0.002 fs was used in the calculations. An electromagnetic pulse (*δ* function) was used for the optical absorption spectrum. Furthermore, there were two main reasons for why the laser intensity used in this work was much higher than that in experiments. First, the laser duration applied here was very short (≈6.6 fs) as compared to the irradiation time (≈1 s) usually used in the experiments, leading to much reduced energy transfer in the former. Second, due to the computational cost, to observe the reduction within 100 fs, a much higher laser intensity was applied reasonably in consideration of the first factor.^[^ ^34^ ^]^ In the simulations, the CO_2_ was initially placed 3.0 Å away from the tip of Ag_20_ and Ag_147_ as shown in Figure 1a and Figure 6a. The laser pulse was a Gaussian wave packet

(3) $$E\left(\omega ,t\right)=E_{\max}\exp\left[-\frac{{\left(t-t_{0}\right)}^{2}}{2\tau^{2}}\right]\cos\left(\omega t-\omega t_{0}+\varphi \right)$$

where the phase *φ* = 0 and *τ* = 3.3 fs. The laser field reaches the maximum *E*_max_ at the time *t*_0_ = 20 fs. laser pulse was also examined with different durations, which were demonstrated to result in the same trends.

## Conflict of Interest

The authors declare no conflict of interest.

## Supporting information

Supporting Information

## Data Availability

Research data are not shared.
