# Supplementary material for: Indirect to Direct Charge Transfer Transition in Plasmon‐Enabled CO2 Photoreduction
Source: Adv Sci (Weinh). 2021 Nov 12;9(2):2102978. doi: 10.1002/advs.202102978 (PMC8805563; doi:10.1002/advs.202102978)
Supplement: Supplementary file 1 — Supporting Information [file ADVS-9-2102978-s001.pdf]

© 2021 Wiley-VCH GmbH

ADVANCED SCIENCE
Open Access

Supporting Information

for *Adv. Sci.,* DOI: 10.1002/advs.202102978

Indirect to Direct Charge Transfer Transition in Plasmon-Enabled CO_2 Photoreduction

Yimin Zhang, Lei Yan, Mengxue Guan, Daqiang Chen, Zhe Xu, Haizhong Guo, Shiqi Hu, Shengjie Zhang, Xinbao Liu, Zhengxiao Guo,* Shunfang Li,* and Sheng Meng.**

Indirect to Direct Charge Transfer Transition in Plasmon-Enabled CO_2 Photoreduction

Yimin Zhang[1,2,4†], Lei Yan[3†], Mengxue Guan[2,4†], Daqiang Chen[2,4], Zhe Xu[1], Haizhong Guo[1*], Shiqi Hu[2,4], Shengjie Zhang[2,4], Xinbao Liu[2,4], Zhengxiao Guo[5,6*], Shunfang Li[1*] and Sheng Meng[2,4*]

[1]*Key Laboratory of Material Physics, Ministry of Education, School of Physics and Microelectronics, Zhengzhou University, Zhengzhou 450001, China*
[2]*Beijing National Laboratory for Condensed Matter Physics and Institute of Physics, Chinese Academy of Sciences, Beijing 100190, China*
[3]*School of Physics and Information Technology, Shaanxi Normal University, Xi'an 710119, China*
[4]*School of Physical Sciences, University of Chinese Academy of Sciences, Beijing 100190, China*
[5]*Departments of Chemistry and Mechanical Engineering, The University of Hong Kong, Hong Kong, China*
[6]*HKU Zhejiang Institute of Research and Innovation, The University of Hong Kong, Hangzhou, China*
*Correspondence authors. Email: hguo@zzu.edu.cn; zxguo@hku.hk; sflizzu@zzu.edu.cn; smeng@iphy.ac.cn
[†]These authors contributed equally to this work.

The supplementary information includes:

Figures S1-S7

Note S1. The influence of adsorption sites

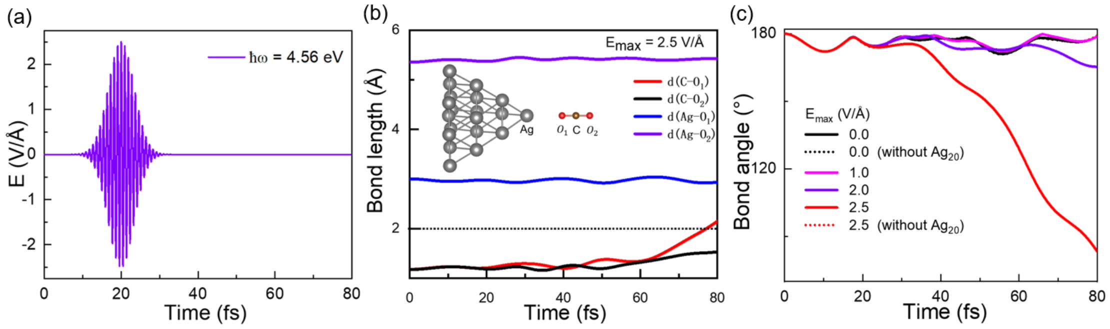

Figure S1. (a) Time evolution of the laser field and (**b**) the bond length of C-O_1, C-O_2, Ag-O_1, and Ag-O_2 with the field strength E_{max} = 2.5V/Å and photon energy $\hbar\omega$ = 4.56 eV. The inset shows the configuration. (c) Time-evolved O-C-O bond angle at different field strength. The dotted lines denote the condition that the Ag cluster is not including in the systems.

Here, Figure S1a denotes the laser pulse applied in our simulation with the field strength E_{max} = 2.5 V/Å and photon energy $\hbar\omega$ = 4.56 eV. Figure S1b displays the time-evolved bond length of C-O_1, C-O_2, Ag-O_1, and Ag-O_2 in the same condition. Figure S1c shows the time-dependent bond angle of ∠O-C-O at different field strength.

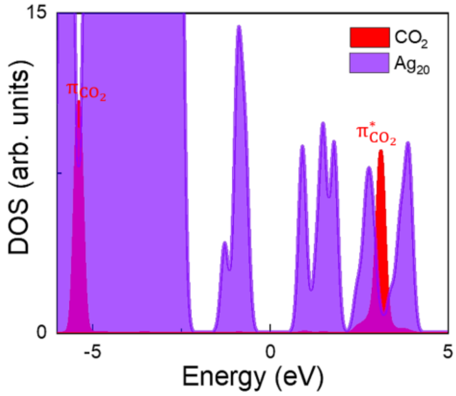

Figure S2. (a) The LDOS of CO_2 and Ag_{20} for the complex.

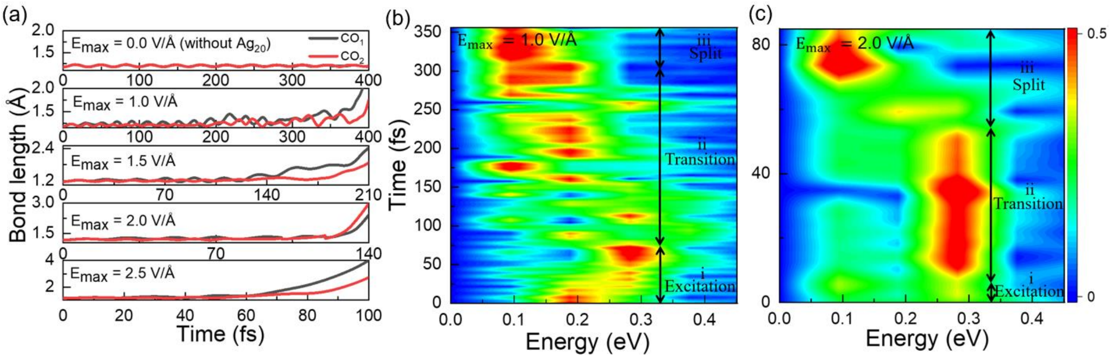

Figure S3. (a) Time-evolved bond length of C-O_1 and C-O_2 at different field strength, with E_{max} = 0.0 V/Å (without Ag_{20}), 1.0 V/Å, 1.5 V/Å, 2.0 V/Å and 2.5 V/Å, respectively. Vibrational modes of the CO_2 species obtained via Fourier transform of the C=O bond length of CO_2 adsorbed on Ag_{20} with (b) E_{max} = 1.0 V/Å, and (c) E_{max} = 2.0 V/Å.

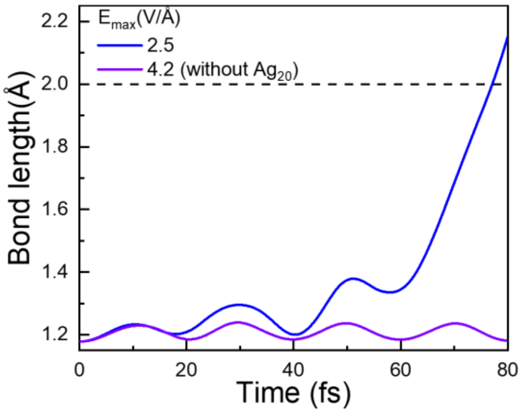

Figure S4. The time-evolved bond length of C=O at E_{max} = 2.5V/Å with Ag_{20} and E_{max} = 4.2 V/Å (corresponds to the FE = 1.7) without $Ag_{20.}$ The black dash line denotes the bond length breaks at 2.0 Å.

Here, Figure S4 describes the time-evolved bond length of C=O at E_{max} = 2.5V/Å with Ag_{20} and E_{max} = 4.2 V/Å (corresponds to the FE = 1.7) without $Ag_{20.}$

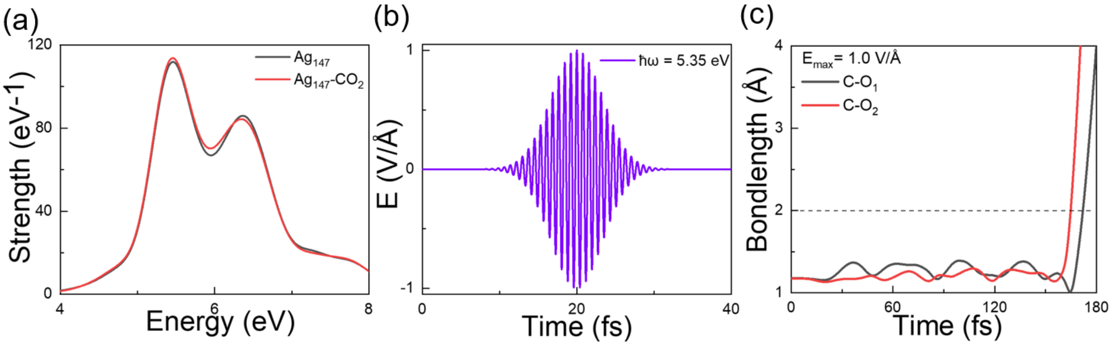

Figure S5. (a) Absorption spectra of Ag_{147} and Ag_{147}-CO_2. Here, three absorption peaks are found, 3.63, 5.35 and 6.34 eV, respectively. However, owing to the relative low absorption strength, the first peak at 3.63 eV is not shown and the highest peak at 5.35 eV is chosen in our simulations. (**b**) Time evolution of the laser field strength and (**c**) bond length of C-O_1 and C-O_2 of the CO_2 under the field strength E_{max} = 1.0 V/Å and photon energy $\hbar\omega$ = 5.35 eV, which shows the complete process of bond breaking.

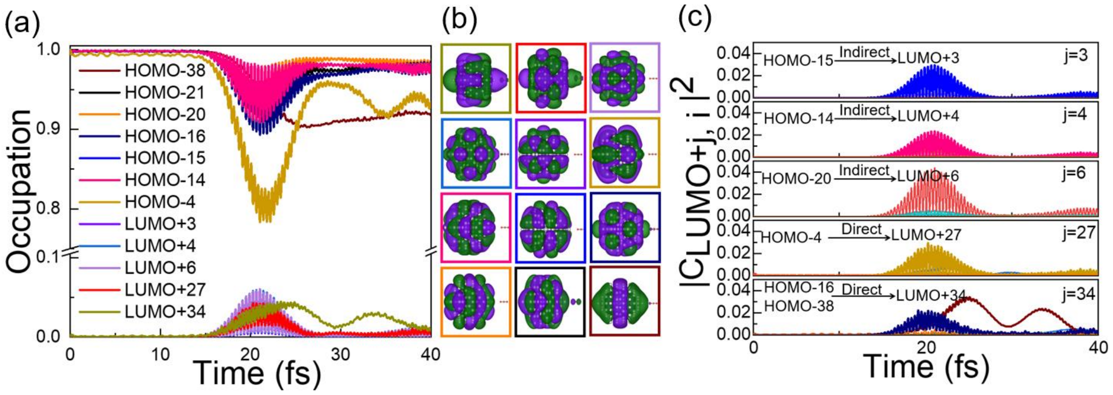

Figure S6. (a) Time-evolved occupation of the twelve Kohn-Sham states and (b) the corresponding wavefunctions shown in boxes of different colors. (c) Time-evolved transition coefficient from the occupied states *i* to LUMO+3, LUMO+4, LUMO+6, LUMO+27 and LUMO+34, respectively. The index *i* (*i* =1-82) denotes the numerical order

of occupied states, while LUMO+j (j = 0-39) corresponds to unoccupied states. A few important orbitals which make major contributions to density changes are labeled.

Note S1. The influence of adsorption sites

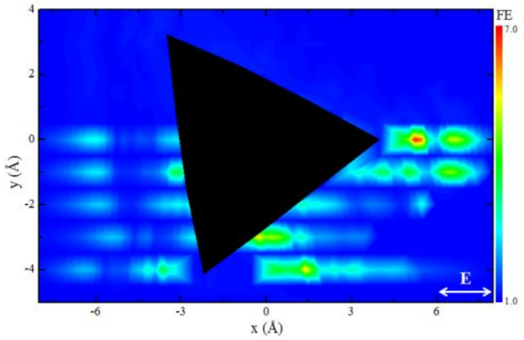

Figure S7. Field enhancement (FE) for the tetrahedron Au_{20}. The laser polarization direction is along the transverse cut going through the tip atom of Au_{20} under the laser pulse with E_{max} = 0.05 V/Å. The data are taken from Ref. [22].

In Yan *et al.*'s work (Ref. [22]) as shown in Fig. S7, we see that the area of largest field enhancement effect is around the tip, analogous to the phenomena of point discharge and the antenna effect, then the edges and surfaces of the tetrahedron. Further, the field enhancement effect could accelerate and strengthen the charge transfer, especially the direct charge transfer when considering the matrix elements of charge transfer processes. Therefore, the tip of Ag_{20} would be the most efficient adsorption site, rather than the edges and facets. As to the icosahedral Ag_{147}, with a configuration close to a sphere, the topological differences among the tip, edge and surface sites are relatively small, and so are the variations of the charge transfer efficiency and the reduction rate.
